# How to evaluate learning in a flipped classroom

**DOI:** 10.3352/jeehp.2018.15.21

**Published:** 2018-09-13

**Authors:** Sun Kim

**Affiliations:** Department of Medical Education, College of Medicine, The Catholic University of Korea, Seoul, Korea; Hallym University, Korea

Recently, with increasing awareness of the limitations of rote learning, which is usually applied in medical education, consensus has emerged that it is important to create an atmosphere where students think for themselves and take initiative in their studies. There are a variety of teaching-learning methods that foster self-directed learning in students. Representative methods that are applied in medical education include problem-based learning, team-based learning, and flipped classroom. Of these teaching-learning methods, flipped classroom has received particular attention with the advent of the era of artificial intelligence. ‘Flipped’ literally means ‘overturned,’ and flipped classroom refers to a class that is administered in a reversed way by changing the conventional class structure. In other words, students learn the topics that will be covered in a class themselves through a video that teachers produce in advance, and then participate in the class with the knowledge that they have acquired. For these purposes, videos should be interesting enough to stimulate students’ curiosity, and less than 15 minutes is generally considered an effective viewing time. Students who attend the class after gaining an understanding of the relevant topics in advance are then able to learn problem-solving and applications in a clinical setting with greater curiosity, instead of focusing on the mere acquisition of knowledge. This helps develop and improve students as learners. Thus, the aims of flipped classroom are to enhance students’ ability to debate various topics, their cooperative learning ability, and most of all, their self-directed learning ability; as such, I believe that all educators who administer classes would agree that such a method is needed. However, learning evaluation—as a way to check how much students’ learning has improved and developed—is not an easy process. Therefore, it is important to develop evaluation methods that are appropriate for specific education methods and to assess their advantages and disadvantages in order to apply them properly. Instead of a best teaching method, it is best to think of there being an optimized teaching-learning method. Likewise, learning evaluations must be optimized based on class content and teaching methods. For instructors, it is an important task to develop and apply an evaluation method in accordance with a teaching method that motivates students to learn medical science and gives them a pleasant experience learning the required content for their major. In other words, an evaluation method that generates learning through its process and fosters a spirit of challenge in students is to be preferred. With this in mind, I suggest the following learning evaluation methods.

First, students watch a video in advance for a flipped classroom and solve some problems that enable self-evaluation. As this is a process in which problems are solved to assess the level of prior learning, the problems need to be composed in a way that enables students to evaluate their learning level themselves. Furthermore, it would be helpful for students to review and re-solve the problems they get wrong. A prior evaluation for preparation is usually applied to team-based learning, where the evaluation is reflected in students’ grades. However, learning evaluation is always a burden to students. Therefore, it is preferable to provide self-evaluation in flipped classroom in a simple form, such as a quiz. Additionally, the meaning of self-evaluation should be explained to students to reduce the perceived burden of grades and to let students know that the self-evaluation is designed to identify their current level, not to affect their ultimate grade.

The second method is peer evaluation, in which students evaluate other students who are participating in the learning process. The importance of peer evaluation for learning has been emphasized by many papers and scholars. This evaluation method is applied in several fields. To efficiently apply peer evaluation in classes, the meaning, necessity, and method of peer evaluation should first be understood. Some principles are prerequisites for peer evaluation, including being on an equal footing, having an open mind, taking responsibility, and facilitating a discussion [1]. To this end, students need some degree of training for peer evaluation, and it is especially important to create an atmosphere without an excessive sense of rivalry and distrust. If a successful peer evaluation is performed when such principles are observed, students can receive very efficient and useful feedback from their peers. Students need to observe their peers’ attitudes toward learning, communication, and cooperation with responsibility and an open mind to evaluate peers on an equal footing, not as rivals, and to provide specific and constructive feedback on that basis. As peer evaluation provides an opportunity for self-reflection to both the evaluators and the evaluated students, it fosters changes in students’ attitude towards learning and helps them experience improvements in learning.

As peer evaluation can be carried out in various ways, it is desirable to apply a method that is appropriate for the purpose of the class. The purpose of flipped classroom is to improve students’ self-directed learning ability and for them to apply their knowledge through problem-solving in clinical cases. Therefore, it is important to use a peer evaluation tool that includes standards such as contributions to problem-solving in their team, cooperative study, and listening. In this context, a valuable experiment could be to evaluate peers qualitatively, instead of merely assigning scores for each standard. Furthermore, students must apply basic principles of feedback and evaluate their peers for each evaluation item. The basic principles of feedback include using descriptive expressions without an evaluative or critical viewpoint, using very specific expressions for certain learning attitudes instead of general phrases, and responding with relevant content that the evaluated peers can accept. It is desirable to foster changes in students’ attitudes toward learning by conducting peer evaluation in flipped classroom as a formative evaluation that is not reflected in grades or credits.

In summary, for classes that aim to develop clinical problem-solving skills based on self-directed learning methods such as flipped classroom, it is recommended to have students conduct self-evaluations motivated by their goals for studying medicine and to apply peer evaluations to foster changes in students’ attitudes toward learning. Although a formative evaluation that is irrelevant to grades, such as self-evaluation or peer evaluation, may not be widely accepted in competitive educational environments such as medicine, such evaluation methods can play an important role in learning methods such as flipped classroom. Therefore, instructors should continuously strive to develop more appropriate evaluation models for their classes.
